# Supplementary material for: Content-rich biological network constructed by mining PubMed abstracts
Source: BMC Bioinformatics. 2004 Oct 8;5:147. doi: 10.1186/1471-2105-5-147 (PMC528731; doi:10.1186/1471-2105-5-147)
Supplement: Additional File 5 — The original Chilibot query results of the term "long-term potentiation (LTP)" and 22 other terms, limiting the latest references analyzed to the years 1990, 1995, 2000, and 2004. [file 1471-2105-5-147-S5.bz2 › chilibotAdditionalFile5/ltp2004/html/LTP_PI-3K.html]

 


 **LTP** and **PI-3K** 
  
Found 6 abstracts in PubMed,  **6 abstracts were retrieved and analyzed**.  


---

 Search Google  |
 PDF files only 
|  EDU domain only 

---

**Interactive relationship** (e.g. stimulation, inhibition, etc)

- However,  **LTP**  recovered after washout of the reversible PI3 kinase  [ **PI-3K** ]  inhibitor LY294002, confirming that  **LTP**  maintenance and expression are distinct events and indicating that PI3 kinase  [ **PI-3K** ]  activity is required for  **LTP**  expression rather than for its maintenance.  Ref: 11978812 J Neurosci, 2002
- two structurally unrelated PI3 kinase  [ **PI-3K** ]  inhibitors, LY294002 and wortmannin, abated established  **LTP** , suggesting that PI3 kinase  [ **PI-3K** ]  is involved in the maintenance expression phase of  **LTP** .  Ref: 11978812 J Neurosci, 2002
- PI3 kinase  [ **PI-3K** ]  and MEK MAP kinase kinase inhibitors had very different effects on  **LTP** , however.  Ref: 12736339 J Neurosci, 2003
- Finally, we found that  **LTP**  induced by theta frequency stimulation was MEK inhibitor insensitive but still PI3 kinase  [ **PI-3K** ]  dependent in hippocampal slices from PSD 95 postsynaptic density 95 mutant mice.  Ref: 12736339 J Neurosci, 2003
- PI3 kinase  [ **PI-3K** ]  inhibitors also block activation of ERK by NMDA receptor stimulation, suggesting that PI3 kinase  [ **PI-3K** ]  inhibitors block  **LTP**  because PI3 kinase  [ **PI-3K** ]  is an essential upstream regulator of ERK activation.  Ref: 12736339 J Neurosci, 2003
- We observed that PI3 kinase  [ **PI-3K** ]  activity was induced in the CA1 region during  **LTP**  of field EPSPs fEPSPs.  Ref: 11978812 J Neurosci, 2002
- Both types of inhibitors suppressed  **LTP**  induced by theta frequency trains of synaptic stimulation, but only PI3 kinase  [ **PI-3K** ]  inhibitors suppressed the induction of  **LTP**  by high frequency stimulation or low frequency stimulation paired with postsynaptic depolarization.  Ref: 12736339 J Neurosci, 2003
- PI 3 kinase  [ **PI-3K** ]  was also activated in response to long term potentiation  [ **LTP** ]   **LTP**  inducing tetanic stimulation.  Ref: 11567621 Neuron, 2001
- Concentrations of PI3 kinase  [ **PI-3K** ]  inhibitors that inhibited  **LTP**  when present during high frequency stimulation had no effect on potentiated synapses when applied after high frequency stimulation, suggesting that PI3 kinase  [ **PI-3K** ]  is specifically involved in the induction of  **LTP** .  Ref: 12736339 J Neurosci, 2003
- Together, our results indicate that the role of PI3 kinase  [ **PI-3K** ]  in  **LTP**  is not limited to its role as an upstream regulator of MAPK signaling but also includes signaling through ERK independent pathways that regulate  **LTP**  induction.  Ref: 12736339 J Neurosci, 2003
- Activation of PI3 kinase  [ **PI-3K** ]  is required for AMPA receptor insertion during  **LTP**  of mEPSCs in cultured hippocampal neurons.  Ref: 12765612 Neuron, 2003
- In this issue of Neuron, Gean and colleagues report the discovery that the PI 3 kinase  [ **PI-3K** ]  AKT PKB pathway contributes to  **LTP**  and the consolidation of amygdala dependent cued fear conditioning in rats.  Ref: 11567607 Neuron, 2001

**Parallel relationship** (e.g. studied together, co-existance, homology, etc.)

- The evidence presented is consistent with the hypothesis that PI 3 kinase  [ **PI-3K** ]  activation, possibly by NGF, plays a role in expression of  **LTP**  in dentate gyrus.  Ref: 10728885 Neuropharmacology, 2000
- In this study, we have investigated the role of PI 3 kinase  [ **PI-3K** ]  in the expression of long term potentiation  [ **LTP** ]   **LTP**  in perforant path granule cell synapses of the rat.  Ref: 10728885 Neuropharmacology, 2000
- To examine this hypothesis, we investigated the effects of PI3 kinase  [ **PI-3K** ]  inhibitors on ERK activation and  **LTP**  induction in the CA1 region of mouse hippocampal slices.  Ref: 12736339 J Neurosci, 2003
- Last, a voltage dependent calcium channel dependent form of  **LTP**  in the CA1 could also be reversibly abated by LY294002, raising the possibility that PI3 kinase  [ **PI-3K** ]  could be required for the expression of multiple forms of synaptic potentiation.  Ref: 11978812 J Neurosci, 2002
- We investigated the role of phosphatidylinositol 3 kinase PI3 kinase  [ **PI-3K** ]  in  **LTP**  at Schaffer collateral commissural fiber CA1 synapses in rat hippocampal slices using biochemical approaches and extracellular electrophysiological recordings.  Ref: 11978812 J Neurosci, 2002
- Inhibitors of both phosphatidylinositol 3 kinase PI3 kinase  [ **PI-3K** ]  and MAPK ERK mitogen activate protein kinase extracellular signal related kinase activation inhibit NMDA receptor dependent long term potentiation  [ **LTP** ]   **LTP** .  Ref: 12736339 J Neurosci, 2003
- PI 3 kinase  [ **PI-3K** ]  inhibitors blocked tetanus induced  **LTP**  as well as PI 3 kinase  [ **PI-3K** ]  activation.  Ref: 11567621 Neuron, 2001
